# Repetitive out of hospital cardiac arrests following pregnancy: a case report of an unfortunate presentation of mitral annular disjunction

**DOI:** 10.1093/ehjcr/ytaa135

**Published:** 2020-05-26

**Authors:** An Van Berendoncks, Jackie McGhie, Hein Heidbuchel, Jolien W Roos-Hesselink

**Affiliations:** y1 Department of Cardiology, Erasmus Medical Centre, Congenital heart disease RG4, PO Box 2040, 3000 CA, Rotterdam, the Netherlands; y2 Department of Cardiology, Antwerp University Hospital, Wilrijkstraat 10, 2650 Edegem, Belgium; y3 Department of Cardiovascular Diseases, University of Antwerp, Universiteitsplein 1, 2610 Wilrijk, Belgium

**Keywords:** Mitral annular disjunction, Pregnancy, Sudden cardiac death, Biplane echocardiography, Case report

## Abstract

**Background:**

Mitral annular disjunction (MAD) is an under-recognized cause of arrhythmic sudden cardiac death, especially in young women. The relation between MAD and the occurrence of arrhythmia during pregnancy has not yet been explored. We would like to stress the importance of careful echocardiographic examination and the vulnerable peripartum period.

**Case summary:**

A 29-year-old woman survived an out of hospital cardiac arrest 4 months after delivery of her first child. The diagnosis was not clear and an implantable cardioverter-defibrillator (ICD) as secondary prevention was implanted. Her second pregnancy and delivery were uneventful. The 12-lead electrocardiogram demonstrated sinus rhythm with right bundle branch block, ventricular extra systoles (premature ventricular contractions), and a right superior axis, i.e. origin in the inferolateral basal left ventricle. Transthoracic 2D echocardiography showed myxomatous mitral valve disease with moderate mitral valve insufficiency with normal left and right heart dimensions and function. However, 4 weeks after delivery she experienced a sudden syncope at home. Implantable cardioverter-defibrillator reading revealed primary ventricular fibrillation, induced by a ventricular premature beat (VPB), terminated with a successful ICD shock. A frame-by-frame echocardiographic analysis of the mitral valve using biplane echocardiographic analysis allowed diagnosis of MAD with detachment of the root of the annulus from the posterolateral ventricular myocardium during systole.

**Conclusion:**

Mitral annular disjunction is an under-recognized cause of arrhythmic sudden cardiac death. Biplane echocardiographic analysis of the mitral annulus can identify MAD and as such may help for risk stratification and sudden cardiac death prevention. Careful follow-up is necessary especially during pregnancy and the postpartum period.

**Learning points**

- Mitral annular disjunction (MAD) is an under-recognized cause of arrhythmic sudden cardiac death, especially in young women.
- Careful frame-by-frame echocardiographic analysis of the mitral annulus with use of biplane may help for risk stratification and sudden cardiac death prevention.
- Women with MAD, planning pregnancy, should receive complete work-up with transthoracic echocardiography, exercise test, 24 h-Holter monitoring and cardiovascular magnetic resonance for adequate risk assessment and need extensive counselling before pregnancy.

## Introduction

Pregnancy is complicated by maternal disease in 1–4% of cases.^1^ Maternal heart disease is the major cause of maternal death during pregnancy in western countries, this includes sudden adult death syndrome, peripartum cardiomyopathy, aortic dissection, myocardial infarction, and ischaemic heart disease.^1^ Tachyarrhythmias are associated with increased maternal mortality risk. In addition to screening for channelopathies, the presence of ventricular arrhythmias warrants further investigation to exclude underlying structural heart disease as this is associated with substantially increased risk of sudden cardiac death for the mother [odds ratio (OR) 40.89, 95% confidence interval (CI) 26.08–64.1; *P* < 0.0001].^1^^,^^2^

Mitral annular disjunction (MAD) is characterized by an abnormal atrial displacement or detachment of the roots of the annulus away from the ventricular myocardium. Mitral annular disjunction has been associated with mitral valve prolapse and malignant ventricular arrhythmias.^3–6^ Only very recently MAD itself has been indicated as an arrhythmic syndrome.^3^ One-third of MAD patients are known to have ventricular arrhythmias and one-tenth severe arrhythmic events. Young age, lower ejection fraction and papillary muscle fibrosis were identified as markers for severe arrhythmias, even without the presence of mitral valve prolapse.^3^ Palpitations are the most frequently reported symptom and premature ventricular contractions (PVCs) are commonly present. The base of the anterior leaflet is normal with only the area under the posterior leaflet being affected in the ventriculo-annular detachment or disjunction.^4^ The individual variation in size of MAD and the interpolated normal, non-disjunctive annulus explains the difficulty in diagnosis.^7^ An experienced eye and frame-by-frame analysis of high-resolution and high-frequency two-dimensional (2D) images are the best ways to diagnose the presence of MAD.^4^

Despite the fact that MAD is considered an under-recognized cause of arrhythmic sudden cardiac death, especially in young women, the relation between MAD and occurrence of arrhythmia during pregnancy has not yet been described. We would like to present a case of a young woman who experienced two episodes of aborted sudden cardiac death in the postpartum period.

The aims of this case report are

1. To include MAD in the differential diagnosis of a young woman suffering from ventricular arrhythmias.
2. To stress the importance of careful echocardiographic analysis and to introduce biplane echocardiographic imaging at the level of the mitral annulus as a useful tool to identify MAD.
3. To draw the attention to a potential increased risk of life-threatening arrhythmia during pregnancy and the postpartum period.

## Timeline

**Table:** 

Date	Events
January 2014	Delivery first child
April 2014	Out of hospital cardiac arrest. Transthoracic echocardiogram: mildly impaired left ventricular (LV) function. DD peripartum or dilated cardiomyopathy implantable cardioverter-defibrillator (ICD) for secondary prevention
February 2016	Cardiac consult 2 weeks pregnantTransthoracic echocardiogram: Myxomatous mitral valve prolapse with moderate insufficiency, normal LV function
October 2016	Delivery second child, uneventful pregnancy, normal LV function
November 2016	Syncope and successful ICD shock, underlying ventricular fibrillation

## Case presentation

A 29-year-old woman with a history of out of hospital cardiac arrest with underlying ventricular fibrillation 4 months after delivery of her first child was referred to our centre to evaluate the risk of a second pregnancy. After her first pregnancy, the differential diagnosis of dilated cardiomyopathy vs. peripartum cardiomyopathy or primary electrical disease was made and an implantable cardioverter-defibrillator (ICD) was implanted as secondary prevention. Extensive investigations, including echocardiography and cardiac magnetic resonance imaging (MRI) revealed no abnormalities apart from mild mitral prolapse and mild left ventricular dysfunction.

**Figure 1:**
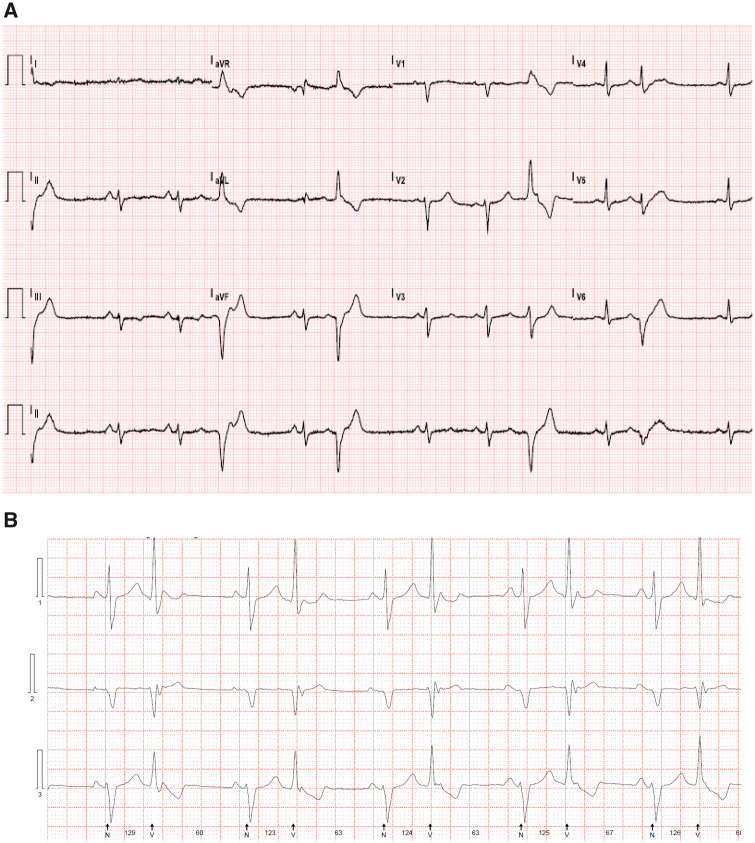
A 12-lead electrocardiogram. (*A*) A 12-lead electrocardiogram registered at routine follow-up shows sinus rhythm with premature ventricular beats with a right bundle branch block and right superior axis morphology, i.e. originating from the left inferolateral basal ventricular wall near the mitral annulus. (*B*) Premature ventricular contraction in bigeminia recorded at 24 h-Holter electrocardiogram.

When she presented in our centre for counselling, she happened to be already 2 weeks pregnant. She had no complaints, no recent history of (pre)syncope’s. Clinical examination showed a lean woman (height 169 cm, weight 59 kg), with normal blood pressure 112/76 mmHg and an oxygen saturation of 98%. On auscultation, an apical mild systolic murmur was present with no signs of heart failure. The 12-lead electrocardiogram (ECG) demonstrated sinus rhythm with right bundle-branch block and ventricular extra systoles (PVC) (*Figure 1*). Exercise-ECG illustrated regular PVCs, no ST-segment changes, and moderate exercise capacity (81% of predicted maximal workload). Echocardiography showed a myxomatous mitral valve with some prolapse and mild to moderate regurgitation with normal left and right ventricular dimensions and function.

Intensive follow-up both at the Department of Gynecology and Cardiology was arranged and the case was repeatedly discussed at the multidisciplinary pregnancy heart team in the presence of cardiologists, gynaecologists, and anaesthesiologists. The maternal risk for cardiac events was categorized as WHO class III and planned delivery with assisted vaginal delivery at our expert centre for pregnancy and cardiac disease was recommended. Medical treatment with metoprolol 100 mg/day was initiated. Prenatal foetal evaluation, genetic screening, and intensive follow-up of foetal growth under treatment with beta-blockers were provided. Pregnancy and delivery were uneventful. A healthy boy, birth weight 3040 g, was born after induction of labour at 39 weeks of pregnancy. She remained in the hospital for rhythm observation for 3 days. No cardiac events occurred in the peripartum period and on transthoracic echo her ventricular function remained good.

**Figure 2:**
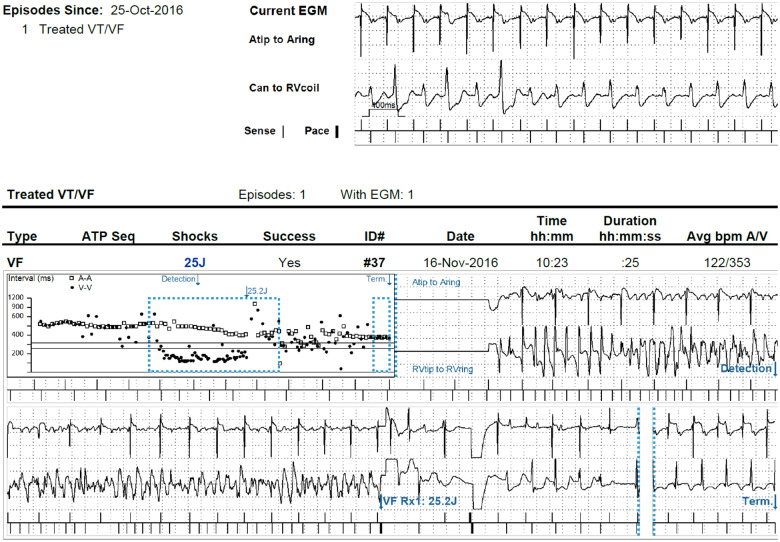
Implantable cardioverter-defibrillator registration during syncope. Implantable cardioverter-defibrillator reading revealed primary ventricular fibrillation, induced by a VPB, terminated with a successful implantable cardioverter-defibrillator shock. The morphology of the inducing beat cannot be determined, since the recording of the intracardiac EGM starts after detection.

However, 4 weeks later, she experienced a sudden syncope at home, while taking a shower. ICD interrogation revealed primary ventricular fibrillation, induced by a premature ventricular beat, terminated with a successful ICD shock. The morphology of the inducing beat could not be determined, since the recording of the intracardiac EGM starts after detection (*Figure 2*). A reversible cause like deterioration of left ventricular function or electrolyte imbalance was not found. Echocardiography revealed no new abnormalities. Medical treatment with metoprolol 100 mg/day was continued.

**Figure 3:**
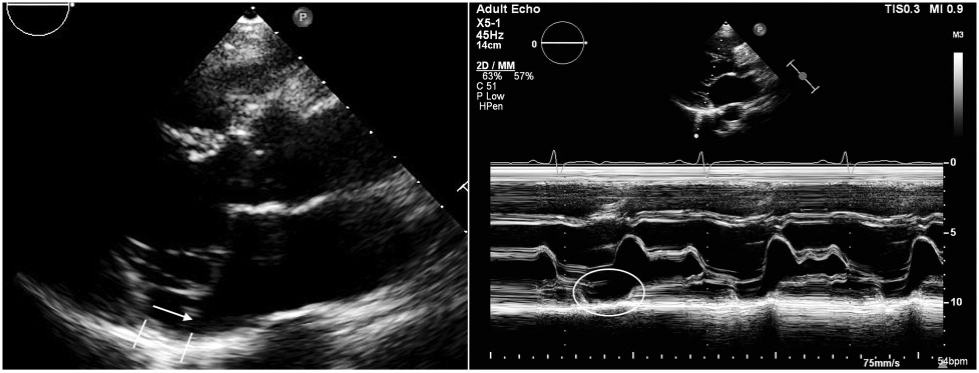
Transthoracic echocardiography. (*A*) Transthoracic echocardiogram showing a parasternal long-axis view illustrating bi-leaflet mitral valve prolapse and mitral annular disjunction diagnosed by the detachment of postero-lateral annulus from the top of the left ventricular posterior wall during end-systole. (*B*) M-mode from the parasternal long-axis view illustrating the end-systolic displacement of the posterior annulus.

She mentioned having PVCs as an adolescent. Reviewing her ECGs showed the presence of regular right bundle-branch block ventricular extra systoles and a right superior axis, i.e. with an origin in the inferolateral basal left ventricle (i.e. close to the posterior mitral annulus) (*Figure 1*). A frame-by-frame echocardiographic analysis allowed the diagnosis of a MAD with detachment of the root of the annulus from the posterolateral ventricular myocardium during systole (*Figure 3*).

Re-evaluation of the cardiac MRI made after her first cardiac arrest confirmed the presence of MAD inferolateral in late systole and detected no signs of fibrosis.

**Figure 4:**
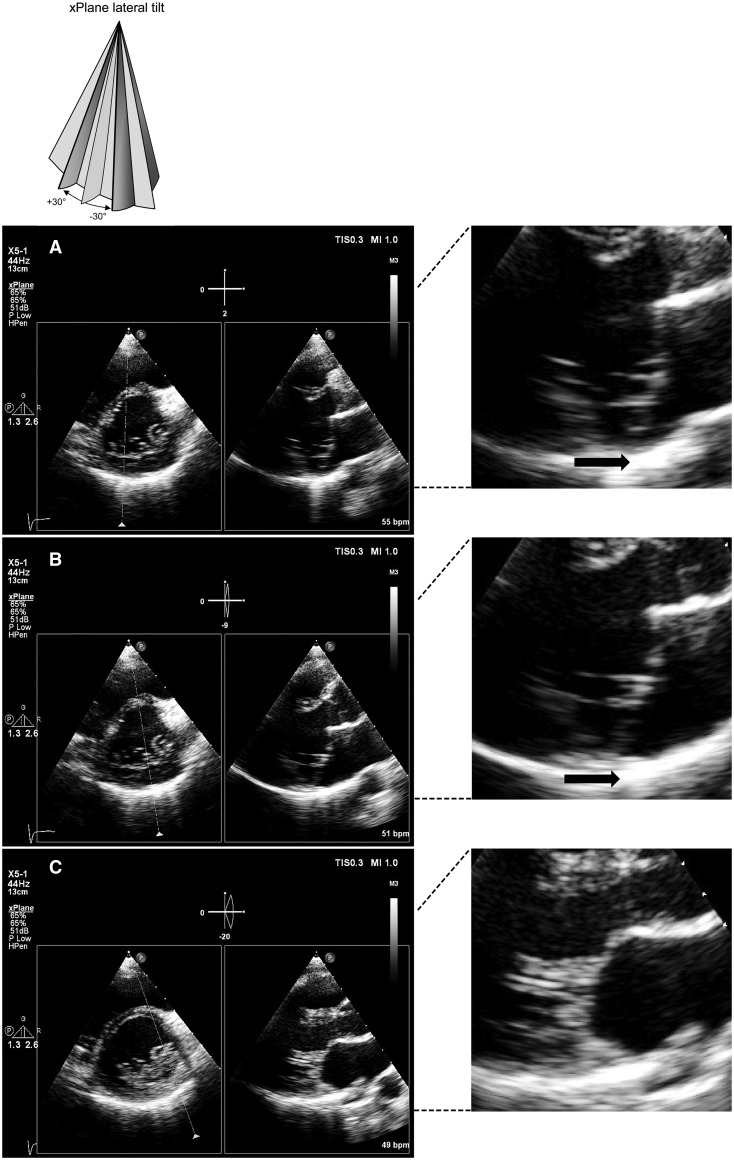
Mitral valve morphology analysis by xPlane with lateral tilt. Careful mitral valve morphology analysis using xPlane imaging from the parasternal short-axis view (primary image with a lateral tilt) and simultaneous orthogonal long-axis views (secondary image) at the mitral valve level. Segmental analysis of the mitral valve annulus illustrates the presence of clear mitral annular disjunction at the posterior annulus, discrete mitral annular disjunction at P2 level and complete absence at the anterior mitral annulus.

*Figure 4* demonstrates an xPlane segmental analysis of the mitral valve and its annulus performed from the parasternal long-axis window with a lateral sweep at mitral annular level, orthogonal short‐axis views are recorded on the secondary image. The xPlane image analysis of the mitral annulus illustrated clearly the presence of MAD at the posterior annulus and a normal anterior mitral annulus, explaining why the diagnosis of MAD can be missed when the cutting plane is incorrect (*Figure 4*).

## Discussion

Mitral annular disjunction is an under-recognized cause of arrhythmic sudden cardiac death, especially in young women. One-tenth of MAD patients have life-threatening arrhythmic events and PVCs are the most common cause for seeking medical advice.^3^ Reviewing the ECGs of our patient we identified since many years, right bundle branch block type PVCs originating from the inferolateral basal left ventricular. The diagnosis of MAD, however, was only made after her second life-threatening arrhythmia on careful transthoracic echocardiogram analysis and re-evaluation of the cardiovascular magnetic resonance (CMR). Recent 3D characterization of MAD using CMR revealed that MAD is only present in up to two-thirds of the mitral ring circumference and longitudinal MAD distance varied considerably.^3^ A few years ago, a new generation matrix transducers became available introducing a new image modality called ‘simultaneous multiplane imaging’ (SMPI). This new modality permits the use of a full electronic rotation of 360° of the 2D image (iRotate) and a simultaneously adjustable biplane (xPlane) 2D image.^8^ The clinical use of this technique in different pathologies, including mitral valve prolapse has been previously described by our group.^8^ Whereas 3D echocardiography requires additional training and often offline analysis, this xPlane imaging is quick, easy to use and may provide unique information on the morphology of the mitral annulus. The xPlane image analysis of the mitral annulus in our case illustrated well the presence of MAD but only at the posterior annulus, explaining why the diagnosis of MAD can be missed as it is so dependent on the position of the cutting plane.

### Role of pregnancy in sudden cardiac death?

Although the presented patient was already known for many years with PVCs and mitral valve prolapse, the only two episodes of sustained ventricular tachycardia (VT) or ventricular fibrillation (VF) occurred in the early postpartum period. Life-threatening VT and VF are very rare during pregnancy or after delivery, but are associated with elevated odds for mortality (OR 40.89, 95% CI 26.08–64.1; *P* < 0.0001) and increased frequencies of maternal and foetal complications.^2^^,^^9^ Arrhythmia has been reported as a complication of mitral valve prolapse during pregnancy,^10^ but as far as we know the relation between MAD and occurrence of arrhythmia during pregnancy has not yet been explored. Perhaps, the so-called ‘mitral valve prolapse syndrome’, which is mentioned in the older literature as being associated with sudden death, was in fact MAD, only not recognized as such. Pregnancy causes not only haemodynamic changes but also the hormonal fluctuations may have impact on the arrhythmogenic risk. It is well-known that women with hereditary long QT syndrome are at substantial increased risk of cardiac events (long QT syndrome-related death, aborted cardiac arrest and syncope) (OR 40.8, 95% CI 3.1–540; *P* = 0.01) especially during the postpartum period.^11^ The continuation of prophylactic treatment with beta-adrenergic blockers not only during pregnancy but also in the postpartum period, at least 40 weeks after delivery is recommended in patients with hereditary long QT and those with catecholaminergic polymorphic VT.^1^^,^^11^ Our patient was very fortunate to have a well-functioning ICD already implanted during the second postpartum period. However, the indication for ICD implantation occurred in the tentative diagnosis of a postpartum cardiomyopathy with VT as recommended in the European Society of Cardiology guidelines.^1^^,^^12^ At the time of presentation in 2016, left ventricular function was completely normal and remained normal during pregnancy and after delivery.

Taking into account the high risk of VT in pregnancy and the high prevalence of arrhythmia in MAD, the indication for prophylactic implantation of an ICD in primary prevention should be further investigated in women with MAD contemplating pregnancy. Medical treatment with beta-blockers was initiated to control the PVCs. However, in the postpartum period, these were not sufficient to prevent a second severe arrhythmic event. One case report describes the disappearance of PVCs after mitral valve replacement.^13^ Therapeutic options for MAD arrhythmias remain to be explored. Genetic studies with the filamin-A subtype suggest that mitral valve disease can be a tissue slippage condition^14^ and probably many more related deficits remain to be detected.^4^ Genetic analysis in our patient revealed a defect in the ldb3 gen, but the clinical relevance is currently still unknown.

According to the recent pregnancy guidelines and based on VT episodes our patient fits the mWHO 3 category, implicating a substantially increased risk of maternal morbidity and mortality.^1^ Careful echocardiographic screening and the intake of beta-blockers also long-term after pregnancy is recommended.

## Conclusion

Presenting this case, we hope to increase the awareness of MAD and the associated risk of arrhythmia particularly in young woman, to highlight the increased risk of arrhythmias during pregnancy and the peripartum period and to contribute to dissemination of the easy to use xPlane echocardiographic analysis for careful posterior mitral annulus examination.

## Lead author biography

**Figure:**
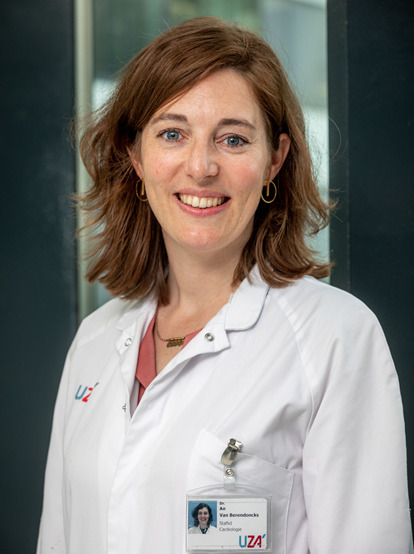


Prof. Dr An Van Berendoncks is a senior staff member at the Department of Cardiology at Antwerp University Hospital since 2014. She completed a fellowship in cardiac rehabilitation in 2017 and combined her staff activities in Antwerp with a part-time fellowship in Congenital Heart Disease at the Thorax Centre, Erasmus MC, Rotterdam, under supervision of Prof Jolien Roos-Hesselink from 2015 to January 2019. She received the European Certificate for Congenital Echocardiography in 2019. Scientifically, she successfully defended her PhD in 2011 on heart failure and cardiac rehabilitation. Currently, she is involved in different research project in GUCH patients. She is a board member of the Belgian Working Group of Congenital Heart Disease.

## Supplementary material

Supplementary material is available at *European Heart Journal - Case Reports* online.

**Slide sets:** A fully edited slide set detailing this case and suitable for local presentation is available online as Supplementary data.

**Consent:** The author/s confirm that written consent for submission and publication of this case report including image(s) and associated text has been obtained from the patient in line with COPE guidance.

**Conflict of interest:** none declared.

## Supplementary Material

ytaa135_Supplementary_Slide-Set
