# Supplementary material for: Repetitive out of hospital cardiac arrests following pregnancy: a case report of an unfortunate presentation of mitral annular disjunction
Source: Eur Heart J Case Rep. 2020 May 26;4(4):1–7. doi: 10.1093/ehjcr/ytaa135 (PMC7501944; doi:10.1093/ehjcr/ytaa135)
Supplement: ytaa135_Supplementary_Slide-Set [file ytaa135_supplementary_slide-set.pptx]

## Slide 1
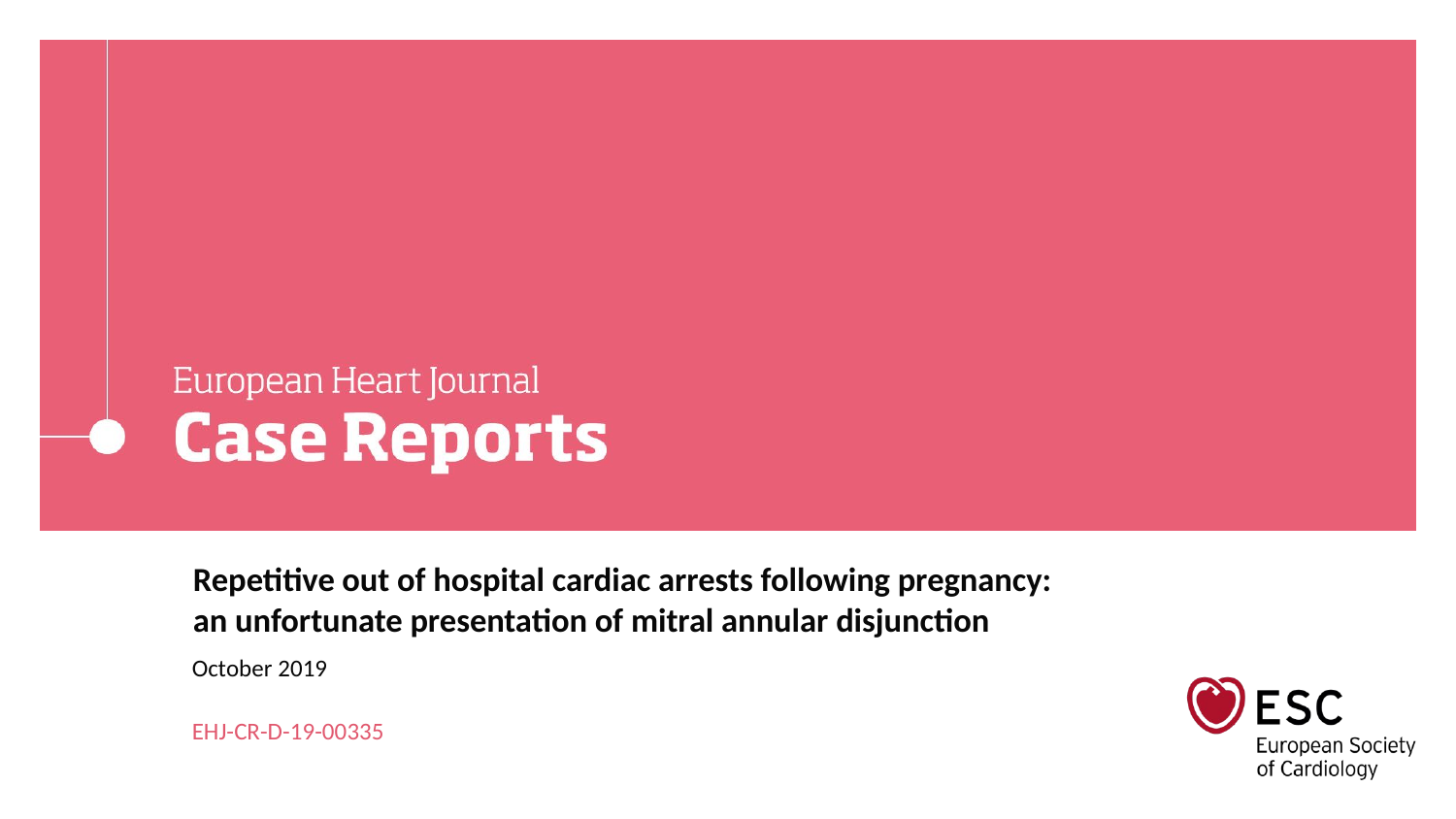

# Repetitive out of hospital cardiac arrests following pregnancy: an unfortunate presentation of mitral annular disjunction
October 2019
EHJ-CR-D-19-00335

## Slide 2
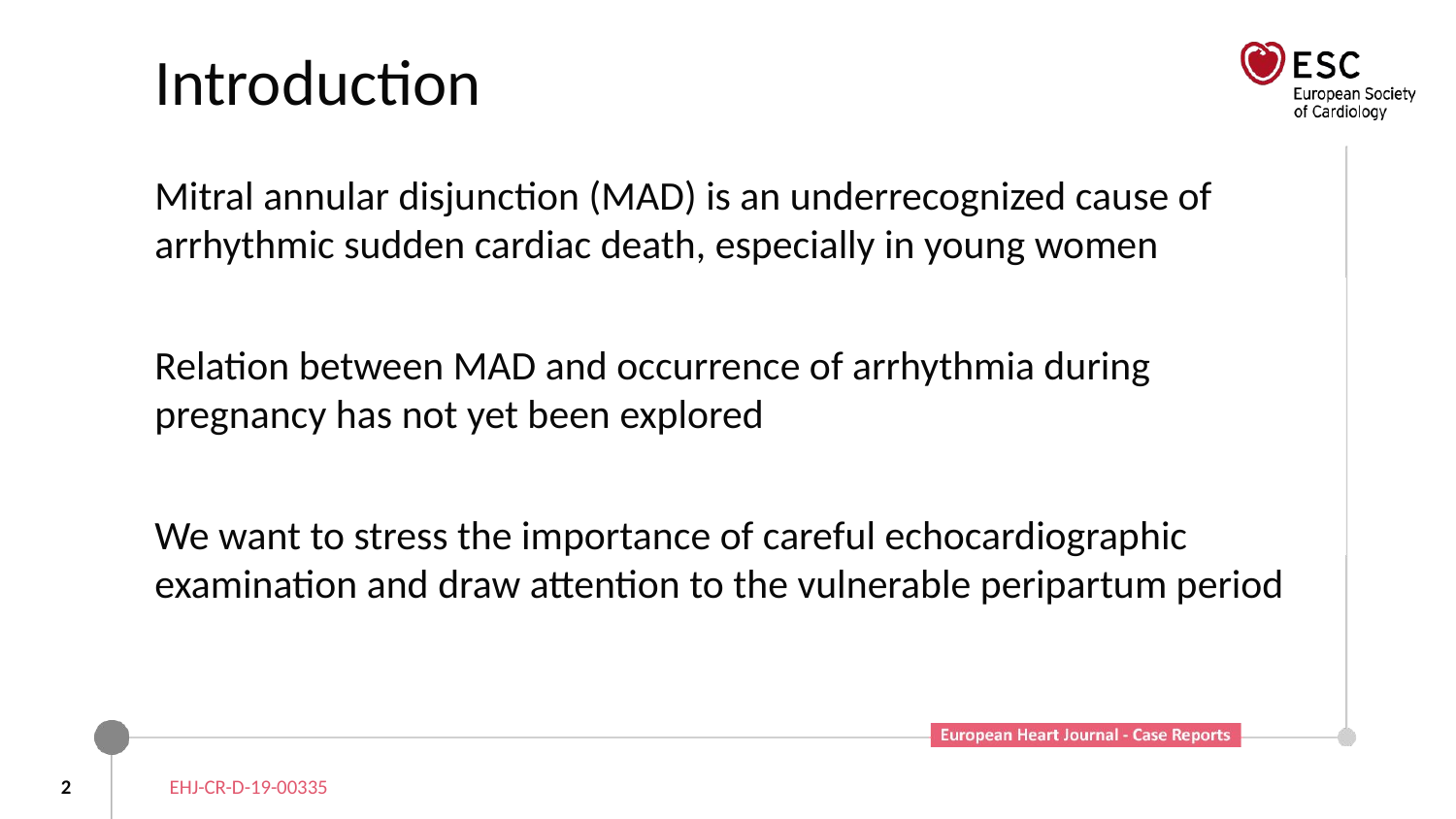

# Introduction
Mitral annular disjunction (MAD) is an underrecognized cause of arrhythmic sudden cardiac death, especially in young women
Relation between MAD and occurrence of arrhythmia during pregnancy has not yet been explored
We want to stress the importance of careful echocardiographic examination and draw attention to the vulnerable peripartum period
2
EHJ-CR-D-19-00335

## Slide 3
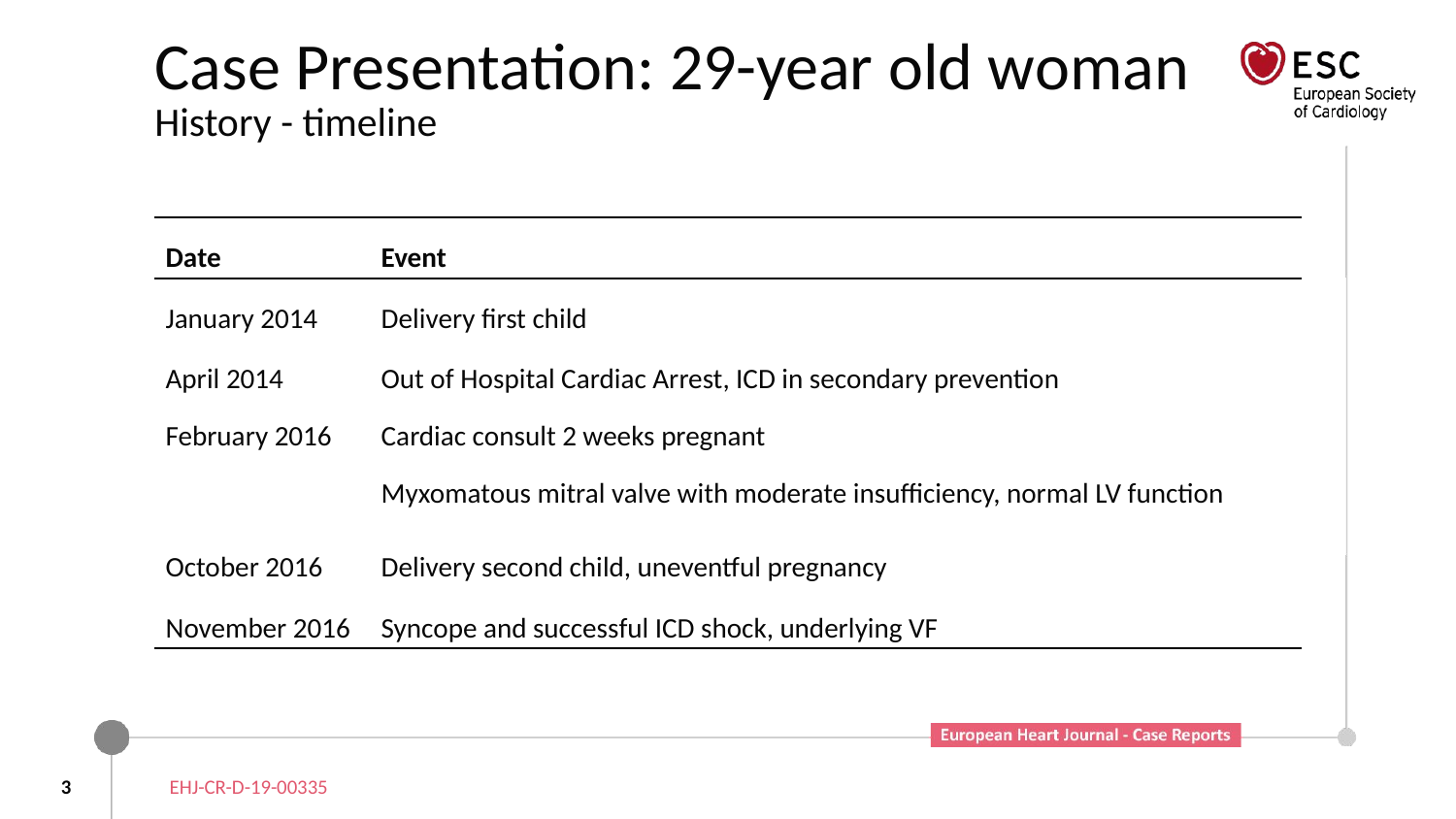

# Case Presentation: 29-year old woman History - timeline
| Date | Event |
| --- | --- |
| January 2014 | Delivery first child |
| April 2014 | Out of Hospital Cardiac Arrest, ICD in secondary prevention |
| February 2016 | Cardiac consult 2 weeks pregnant Myxomatous mitral valve with moderate insufficiency, normal LV function |
| October 2016 | Delivery second child, uneventful pregnancy |
| November 2016 | Syncope and successful ICD shock, underlying VF |
3
EHJ-CR-D-19-00335

## Slide 4
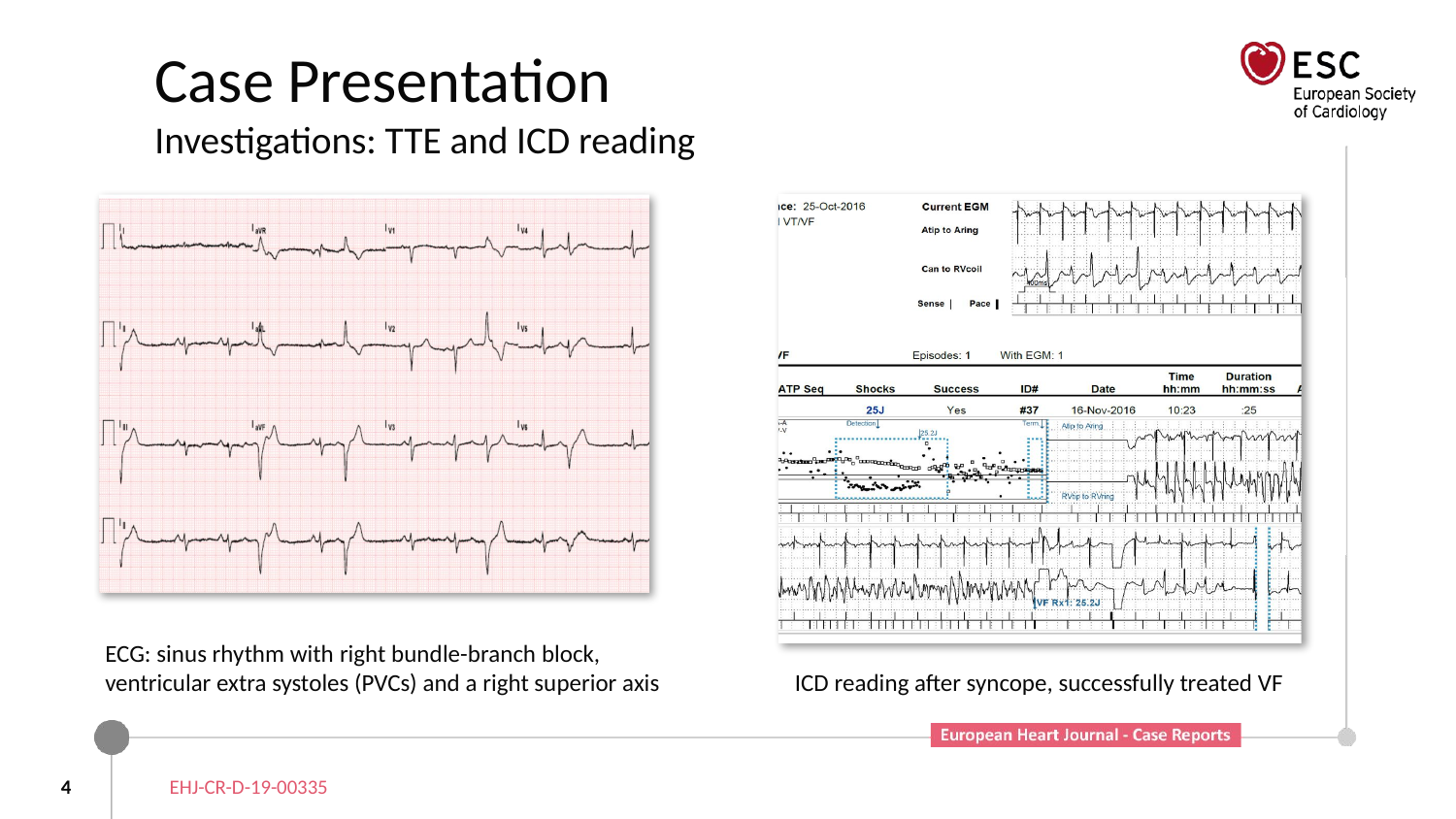

# Case Presentation Investigations: TTE and ICD reading
ECG: sinus rhythm with right bundle-branch block,
ventricular extra systoles (PVCs) and a right superior axis
ICD reading after syncope, successfully treated VF
4
EHJ-CR-D-19-00335

## Slide 5
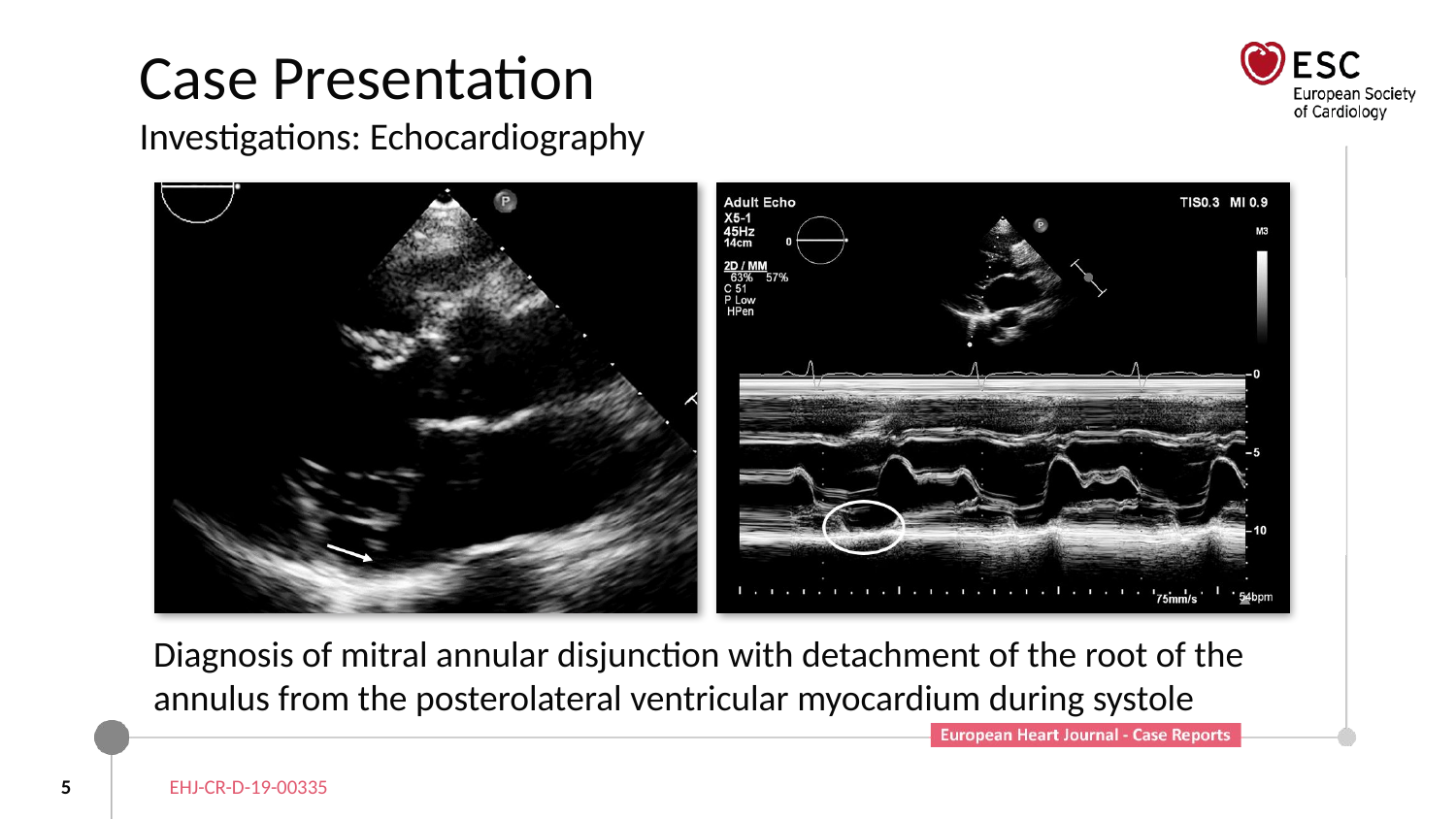

# Case Presentation Investigations: Echocardiography
Diagnosis of mitral annular disjunction with detachment of the root of the annulus from the posterolateral ventricular myocardium during systole
5
EHJ-CR-D-19-00335

## Slide 6
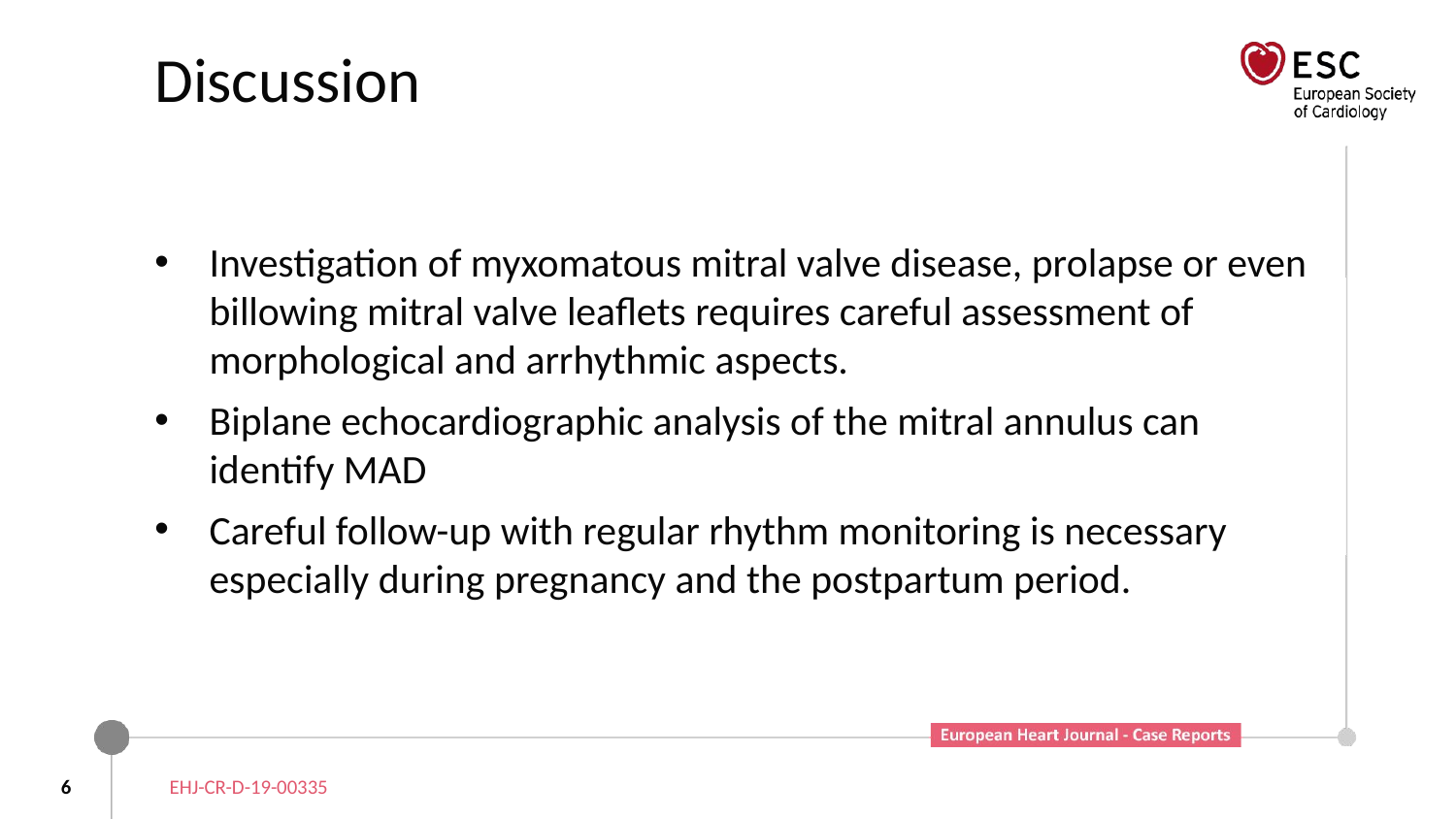

# Discussion
Investigation of myxomatous mitral valve disease, prolapse or even billowing mitral valve leaflets requires careful assessment of morphological and arrhythmic aspects.
Biplane echocardiographic analysis of the mitral annulus can identify MAD
Careful follow-up with regular rhythm monitoring is necessary especially during pregnancy and the postpartum period.
6
EHJ-CR-D-19-00335

## Slide 7
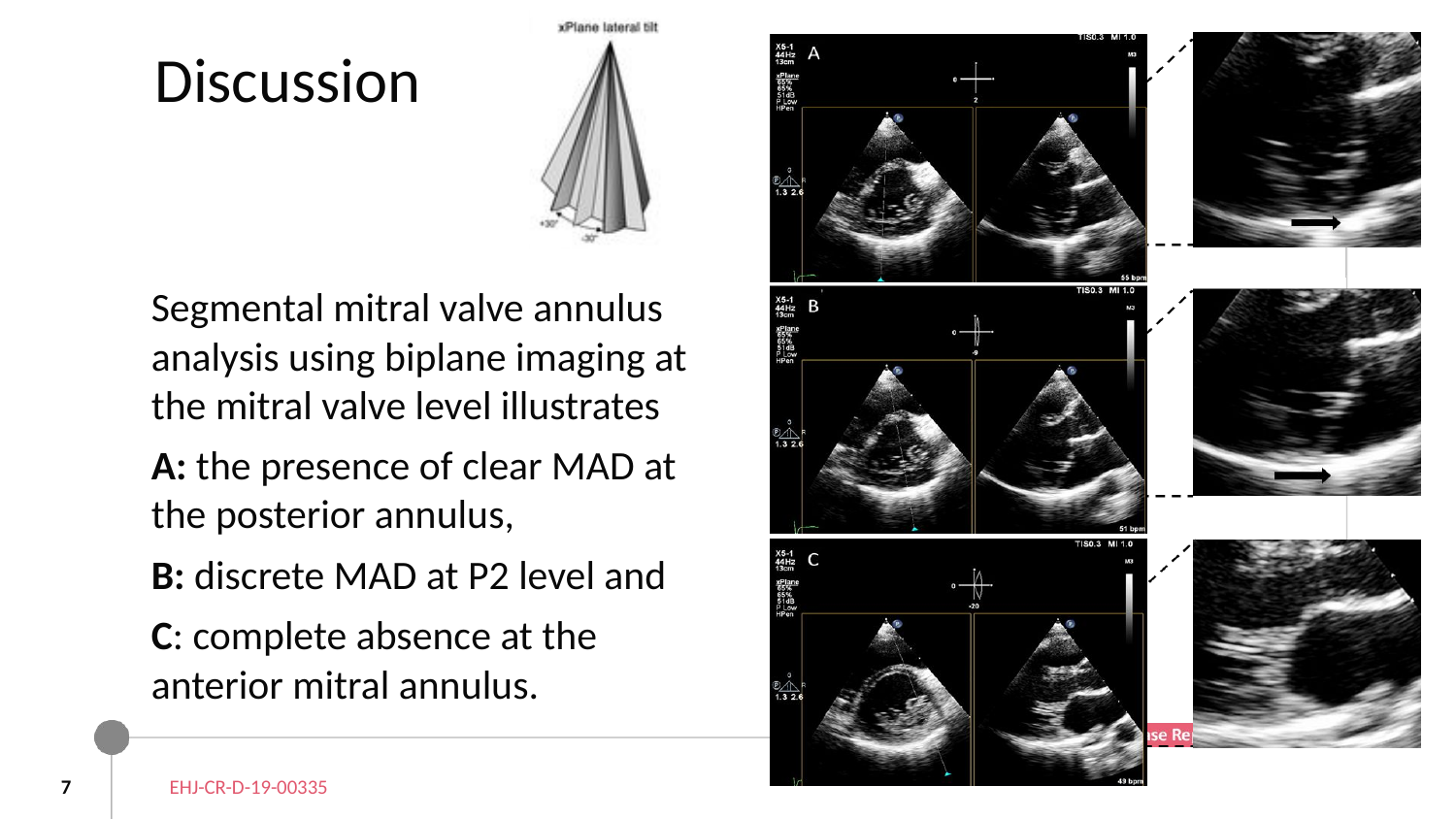

# Discussion
Segmental mitral valve annulus analysis using biplane imaging at the mitral valve level illustrates
A: the presence of clear MAD at the posterior annulus,
B: discrete MAD at P2 level and
C: complete absence at the anterior mitral annulus.
7
EHJ-CR-D-19-00335

## Slide 8
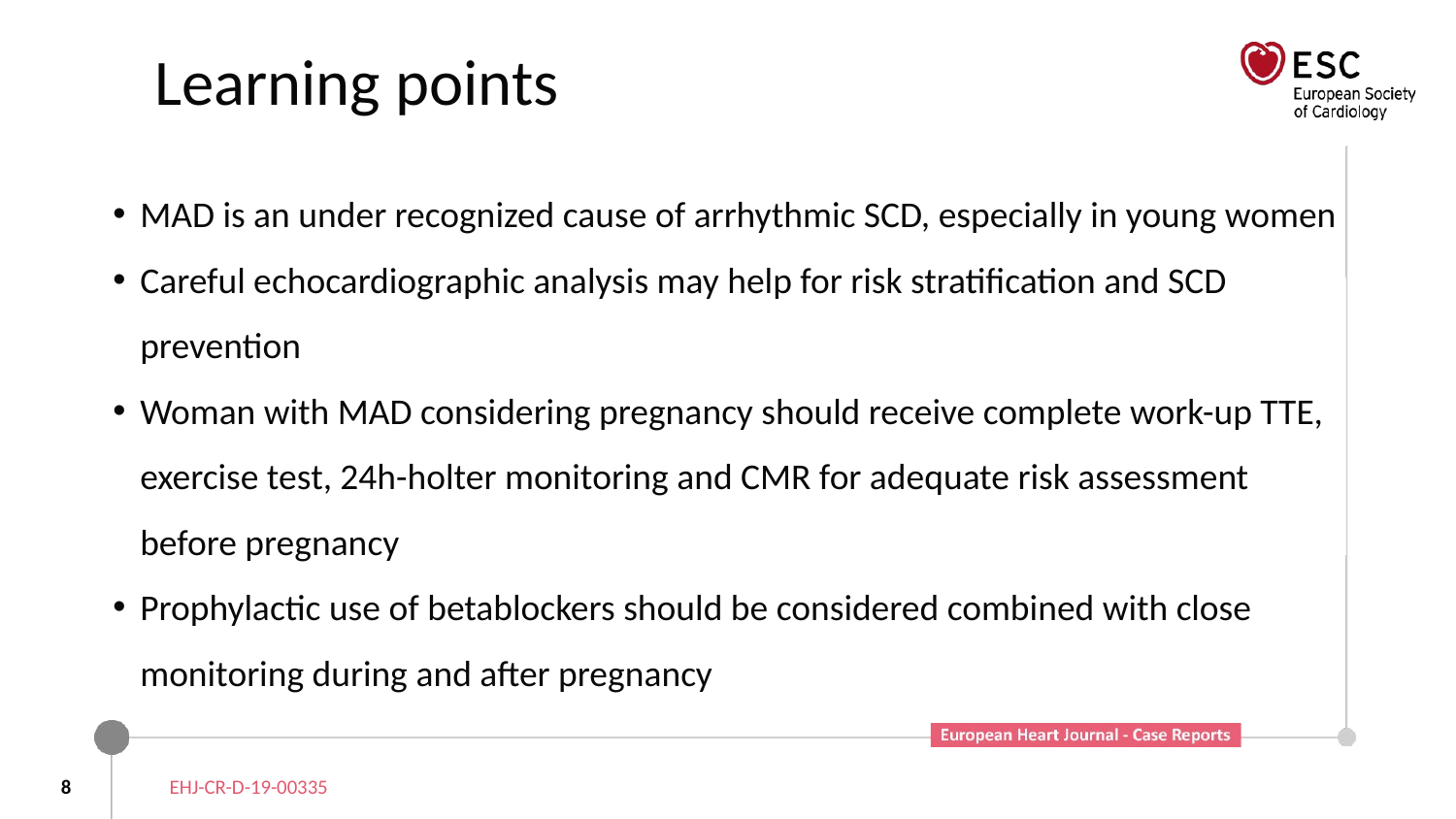

# Learning points
MAD is an under recognized cause of arrhythmic SCD, especially in young women
Careful echocardiographic analysis may help for risk stratification and SCD prevention
Woman with MAD considering pregnancy should receive complete work-up TTE, exercise test, 24h-holter monitoring and CMR for adequate risk assessment before pregnancy
Prophylactic use of betablockers should be considered combined with close monitoring during and after pregnancy
8
EHJ-CR-D-19-00335

## Slide 9
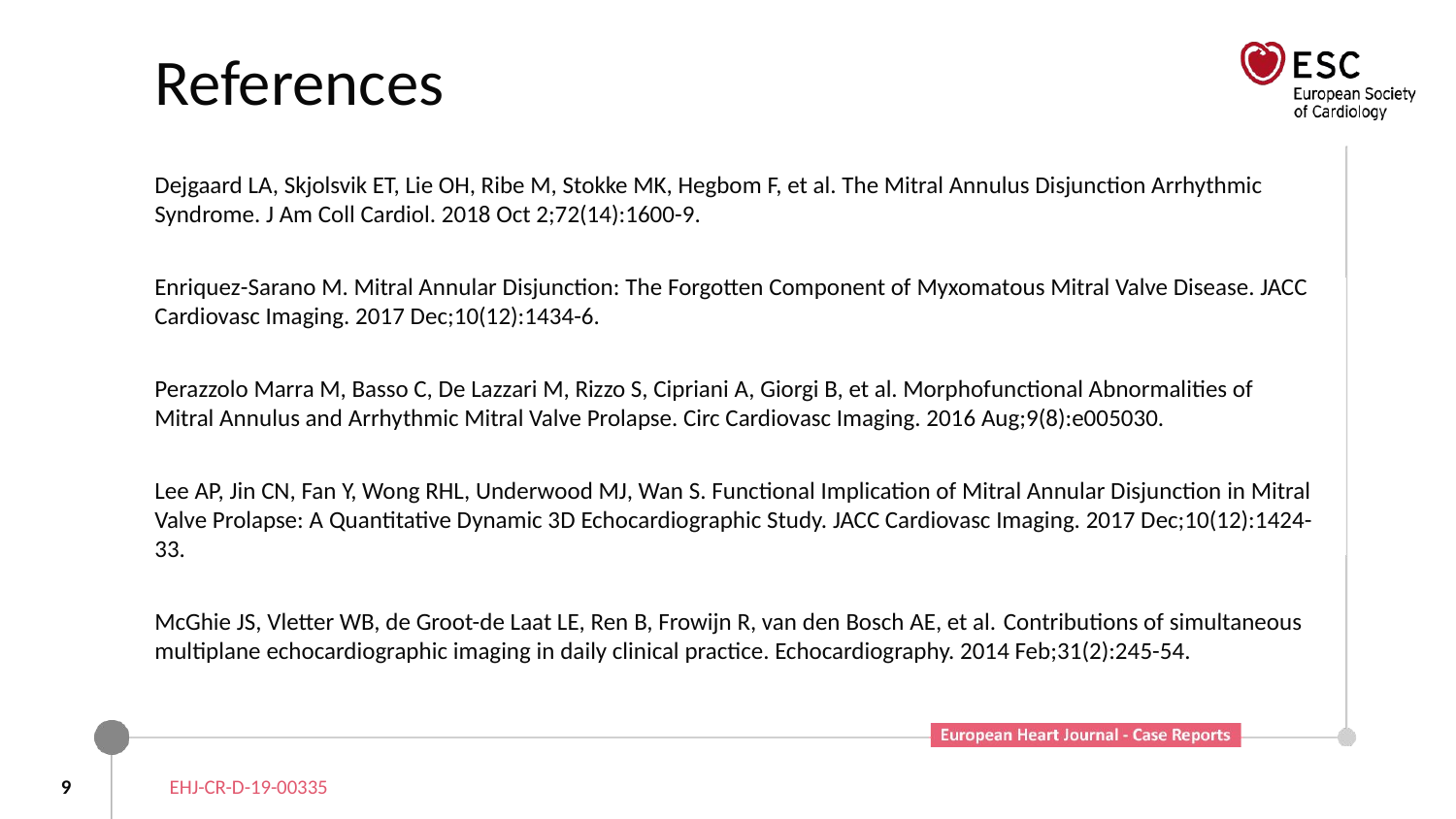

# References
Dejgaard LA, Skjolsvik ET, Lie OH, Ribe M, Stokke MK, Hegbom F, et al. The Mitral Annulus Disjunction Arrhythmic Syndrome. J Am Coll Cardiol. 2018 Oct 2;72(14):1600-9.
Enriquez-Sarano M. Mitral Annular Disjunction: The Forgotten Component of Myxomatous Mitral Valve Disease. JACC Cardiovasc Imaging. 2017 Dec;10(12):1434-6.
Perazzolo Marra M, Basso C, De Lazzari M, Rizzo S, Cipriani A, Giorgi B, et al. Morphofunctional Abnormalities of Mitral Annulus and Arrhythmic Mitral Valve Prolapse. Circ Cardiovasc Imaging. 2016 Aug;9(8):e005030.
Lee AP, Jin CN, Fan Y, Wong RHL, Underwood MJ, Wan S. Functional Implication of Mitral Annular Disjunction in Mitral Valve Prolapse: A Quantitative Dynamic 3D Echocardiographic Study. JACC Cardiovasc Imaging. 2017 Dec;10(12):1424-33.
McGhie JS, Vletter WB, de Groot-de Laat LE, Ren B, Frowijn R, van den Bosch AE, et al. Contributions of simultaneous multiplane echocardiographic imaging in daily clinical practice. Echocardiography. 2014 Feb;31(2):245-54.
9
EHJ-CR-D-19-00335
